# Alteration of the Canine Metabolome After a 3-Week Supplementation of Cannabidiol (CBD) Containing Treats: An Exploratory Study of Healthy Animals

**DOI:** 10.3389/fvets.2021.685606

**Published:** 2021-07-16

**Authors:** Elizabeth M. Morris, Susanna E. Kitts-Morgan, Dawn M. Spangler, Ibukun M. Ogunade, Kyle R. McLeod, David L. Harmon

**Affiliations:** ^1^Department of Animal and Food Sciences, University of Kentucky, Lexington, KY, United States; ^2^College of Veterinary Medicine, Lincoln Memorial University, Harrogate, TN, United States; ^3^Division of Animal and Nutritional Science, West Virginia University, Morgantown, WV, United States

**Keywords:** cannabidiol, canine, metabolomics, biomarkers, metabolites

## Abstract

Despite the increased interest and widespread use of cannabidiol (CBD) in humans and companion animals, much remains to be learned about its effects on health and physiology. Metabolomics is a useful tool to evaluate changes in the health status of animals and to analyze metabolic alterations caused by diet, disease, or other factors. Thus, the purpose of this investigation was to evaluate the impact of CBD supplementation on the canine plasma metabolome. Sixteen dogs (18.2 ± 3.4 kg BW) were utilized in a completely randomized design with treatments consisting of control and 4.5 mg CBD/kg BW/d. After 21 d of treatment, blood was collected ~2 h after treat consumption. Plasma collected from samples was analyzed using CIL/LC-MS-based untargeted metabolomics to analyze amine/phenol- and carbonyl-containing metabolites. Metabolites that differed — fold change (FC) ≥ 1.2 or ≤ 0.83 and false discovery ratio (FDR) ≤ 0.05 — between the two treatments were identified using a volcano plot. Biomarker analysis based on receiver operating characteristic (ROC) curves was performed to identify biomarker candidates (area under ROC ≥ 0.90) of the effects of CBD supplementation. Volcano plot analysis revealed that 32 amine/phenol-containing metabolites and five carbonyl-containing metabolites were differentially altered (FC ≥ 1.2 or ≤ 0.83, FDR ≤ 0.05) by CBD; these metabolites are involved in the metabolism of amino acids, glucose, vitamins, nucleotides, and hydroxycinnamic acid derivatives. Biomarker analysis identified 24 amine/phenol-containing metabolites and 1 carbonyl-containing metabolite as candidate biomarkers of the effects of CBD (area under ROC ≥ 0.90; *P* < 0.01). Results of this study indicate that 3 weeks of 4.5 mg CBD/kg BW/d supplementation altered the canine metabolome. Additional work is warranted to investigate the physiological relevance of these changes.

## Introduction

Cannabidiol (CBD) is one of over 100 phytocannabinoids produced by glandular trichomes of *Cannabis sativa* (1–3). Cannabidiol has been proposed to induce a plethora of beneficial health effects, including anxiolytic, antipsychotic, anti-inflammatory, analgesia, and immunomodulatory effects (4, 5). The wide range of potential therapeutic effects are thought to be a result of multiple mechanisms of action on receptors known to be a part of the endocannabinoid system [ECS; (6, 7)]. Due to the psychoactive effects caused by the action of Δ9-tetrahydrocannabinol (THC) on the CB1 receptor, hemp and all its products, including CBD, were classified as illegal, Schedule I drugs under the Controlled Substances Act (CSA) in 1970 (8, 9). This severely restricted access to CBD as well as the potential for research into the effects of CBD on mammalian physiological systems. As a result, there was little to no opportunity to investigate the potential effects of CBD until industrial hemp was removed from the CSA and CBD was removed from the Schedule I drug list in 2018 (10).

Despite the lack of research, public perception of the supposed health benefits of CBD has led to a rapid expansion of the market for industrial hemp-derived CBD products. In one survey of over 1,100 dog owners, 79.8% said they had purchased hemp or marijuana for their dogs for reasons such as pain relief, relieving anxiety or noise phobia, and reduction of inflammation (11). While this survey represents only a small portion of the population, it does demonstrate favorable perceptions of pet owners on the safety and efficacy of CBD use in pets. Yet despite public opinion, there remains a substantial lack of scientific literature to back these anecdotal claims, particularly regarding potential effects of CBD on long-term health and safety, which makes continued research into its potential benefits and risks all the more essential.

Mass spectrometry-based metabolomics has been increasingly used to assess the health and status of animals and to analyze metabolic alterations caused by diet, disease, or other factors (12–14). Targeted metabolomics can be used to quantify defined groups of metabolites, whereas untargeted metabolomics provides a comprehensive analysis of all measurable analytes in a sample, including any unknowns (15, 16). In instances where the specific metabolites of interest are unknown, untargeted metabolomics can also be used to discover specific biomarkers for later use in targeted metabolomics studies as well as pathway analysis (17, 18). In recent years, chemical isotope labeling (CIL) and liquid chromatography-mass spectrometry (LC-MS)-based untargeted metabolomics has provided an opportunity to analyze metabolites based on chemical groups, including metabolites containing the amine/phenol chemical group (amine/phenol metabolome) which are common intermediate products of amino acid metabolism, and metabolites containing a carbonyl group (carbonyl-metabolome) which common intermediate products of energy metabolism (19).

To date, there has been no evaluation of the effect of CBD on the canine metabolome. Therefore, the objective of this exploratory study was to evaluate the impact of CBD supplementation on the canine metabolome with the underlying hypothesis that after 3 weeks of supplementation, CBD would alter the canine metabolome compared with control. This was accomplished through the use of untargeted metabolomics and biomarker analysis of amine/phenol- and carbonyl-containing metabolites.

## Materials and Methods

This study was approved by the Lincoln Memorial University (LMU) institutional animal care and use committee (protocol 1911-RES) before the start of the study. All housing and husbandry were provided in accordance with the Animal Welfare Act, the Guide for the Care and Use of Laboratory Animals (8th ed.), and all applicable LMU protocols.

### Subjects and Diets

Sixteen dogs (eight male, eight female, 9 months to 4 years of age, 18.2 ± 3.4 kg BW) of various mixed breeds were received from a local shelter for inclusion in this study. Additionally, the shelter was informed of and gave consent for the use of the dogs for research purposes before their arrival. Prior to beginning the experiment, each dog had a complete blood count (CBC) and serum chemistry analysis (IDEXX Laboratories, Inc., Westbrook, Maine) performed, along with physical evaluation by the attending veterinarian and a fecal examination to rule out any underlying disease that might preclude enrollment. Dogs were excluded if they demonstrated serious behavioral issues, such as human aggression that would endanger research personnel, were severely emaciated, classified as a body condition score <3.5 on a 9-point scale (where one is emaciated and 9 is obese), or if their initial evaluations revealed an underlying disease that required more than routine treatments (such as heartworm positive dogs).

Dogs were individually housed in 1.2 × 1.8 m cages within one of two dog kennels at the LMU DeBusk Veterinary Teaching Center. They were stratified by treatment and sex and evenly distributed between the two kennels. Dogs were fed Purina Pro Plan EN Gastroenteric Fiber Balance Dry Dog Food (Nestle Purina, Inc., St. Louis, MO) to meet the daily metabolizable energy requirements of neutered adult dogs at maintenance, calculated as (70 ^*^ BW^0.75^) ^*^ 1.6 and split into two meals per day. Body weight and body condition score (5-point scale) were assessed weekly for the adjustment of diets. Dogs arrived from the shelter and were started on the study diet more than 37 days prior to starting treatments and 58 days before collecting samples for this study.

### Experimental Design and Treatments

These dogs were participating in a concurrent study evaluating the impact of CBD on canine voluntary activity (20) with treatments consisting of 0 (placebo treats; CON) or 75.6 ± 5.86 mg CBD/d (CBD). Dogs were blocked by baseline activity before being stratified by age, weight, and sex and randomly assigned to treatments within each block. The CBD was a constituent of a proprietary industrial hemp extract (AgTech Scientific, Paris, KY) that was incorporated into treats and administered in the form of 2 treats daily, each containing half the daily dose. Both CON and CBD treats were composed of the following ingredients: chicken, chicken liver, Asian carp, catfish, and in the case of the CBD treats, industrial hemp extract. Cannabidiol was the primary constituent of the industrial hemp extract; however, trace THC was present in the CBD treatment (2.9 ± 0.22 mg THC/d). Based on the mean BW of dogs included in the study and analysis of the treats, mean dose of CBD ± SD was 4.5 ± 0.77 mg CBD/kg BW/d. Treats were offered solely as a reward upon kennel re-entry following twice-daily exercise within 30 min of meals.

### Blood Sample Collection

After 21 d of treatment administration, ~6 mL of blood was collected *via* cephalic catheter or jugular venipuncture ~2 h after the final treat administration. The selection of this time point was based on previous work demonstrating the half-life of elimination of CBD to be between 1 and 4 h after oral administration (21–23). Blood samples were collected into tubes containing sodium heparin and were immediately centrifuged at 1,645 × g for 10 min. Plasma was collected after centrifugation then stored at −20°C (<12 h) before long-term storage at −80°C.

### CIL/LC-MS-Based Untargeted Metabolomics Analysis

Untargeted metabolomic profiling was done using a CIL/LC-MS-based technique with an Agilent 1100 LC system (Palo Alto, CA) connected to a Bruker Impact HD quadrupole time-of-flight (QTOF) MS (Billerica, MA). This technique uses a differential isotope labeling (^12^C and ^13^C-labeling) to separate metabolites based on chemical groups followed by LC-MS analysis (19). Detailed information regarding sample preparation, labeling, normalization, LC-UV and LC-MS setup, and metabolite quantification have been reported elsewhere (24, 25). Typical coefficient of variation for this high-performance chemical isotope labeling LC-MS method for metabolome analysis is in the range of 5–10% for individual metabolites (26–28). In this study, amine/phenol- and carbonyl-containing metabolites were analyzed. A total of 19 LC-MS data files were generated (three quality control samples, eight CBD samples, and eight CON samples).

### Metabolite Data Processing

Raw data processing on the 19 LC-MS data files was performed using ISOMS Pro 1.0 according to procedures described by Mung and Li (25). Peak pairs whose mean (sample) / mean (blank) was ≤ 4.0 were filtered out. Peak pairs with no data present in at least 80% of the samples were filtered out. The final metabolite-intensity table was generated using IsoMS-Quant (29).

### Metabolite Identification

A two-tier identification approach was used to perform metabolite identification. In tier 1, peak pairs were searched against a chemical isotope-labeled metabolite library (CIL Library) based on accurate mass and retention time (29). The CIL Library contains 1,213 experimental entries, including 711 amines/phenols and 90 carbonyls. In tier 2, a linked identity library (LI Library) was used for the identification of the remaining peak pairs. The LI Library includes over 2,000 human endogenous metabolites from 68 metabolic pathways, providing high-confidence putative identification results based on accurate mass and predicted retention time matches (30).

### Statistical Analysis

The final metabolite intensity tables for the amine/phenol- and carbonyl-containing metabolome were imported separately into MetaboAnalyst 5.0 software package [www.metaboanalyst.ca; (31)] for statistical analysis. Before statistical analysis, the data were log-transformed, normalized by the median, and auto-scaled. Median scaling was performed to eliminate unwanted inter-sample variations to make the individual samples more comparable to each other. Auto-scaling was used to make the metabolites more comparable in magnitude to each other. Univariate (volcano plot) and multivariate analysis (Partial least squares discriminant analysis [PLS-DA] scores plot) were then generated to identify overall treatment differences across the multivariate dataset. The volcano plot was constructed by plotting the fold change (FC; CBD/CON) of each metabolite against its *P*-value. Metabolites with FC ≥ 1.2 or ≤ 0.83 having a false discovery ratio (FDR) ≤ 0.05 were considered to be differentially increased or decreased relative to CON, respectively. The utility of the metabolites with FC ≥ 1.2 or ≤ 0.83 and FDR ≤ 0.05 to serve as potential biomarkers of the effects of CBD was tested using a receiver operating characteristic (ROC) curves as calculated by the ROCCET web server using MetaboAnalyst 5.0 software package. Metabolites with an area under ROC (AUROC) ≥ 0.90 and a *P* ≤ 0.05 were considered excellent biomarkers as defined in Xia et al. (17).

## Results

### Amine/Phenol Metabolites

**Figure 1:**
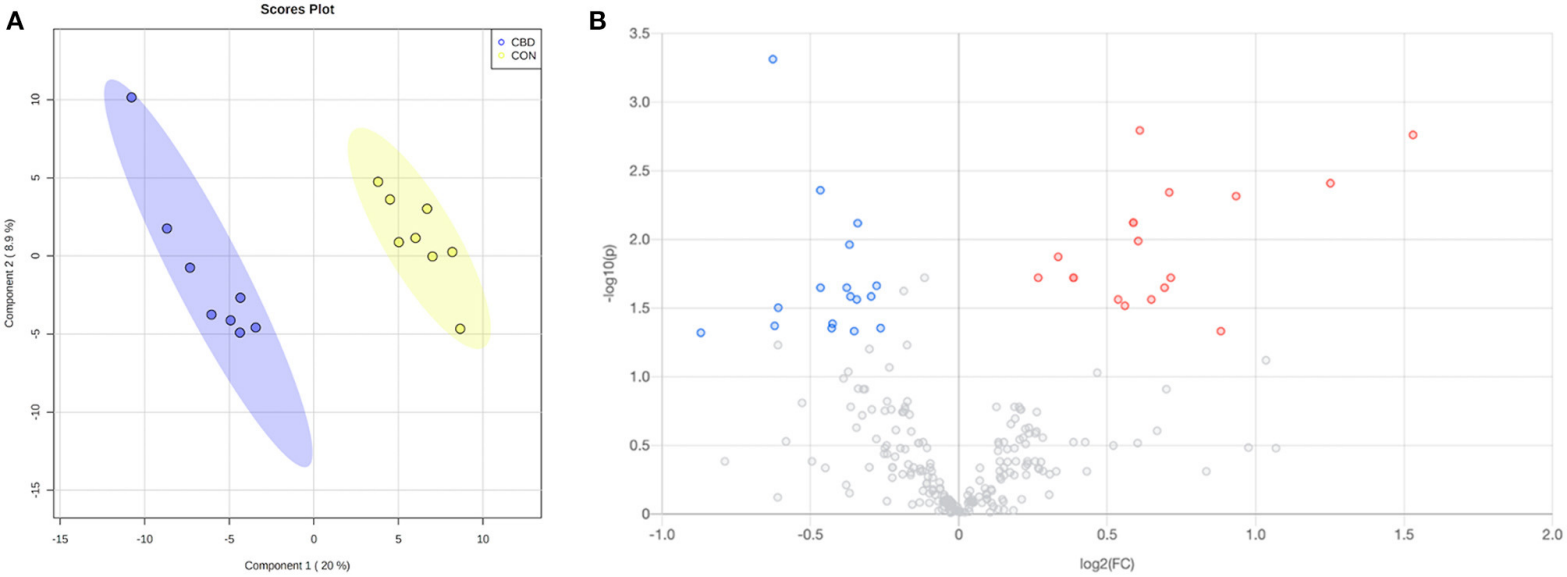
**(A)** Partial least squares discriminant analysis (PLS-DA) scores plot and **(B)** volcano plot showing the differential amine/phenol-containing metabolites. Fold change (FC) ≥ 1.2 (in red) or ≤ 0.83 (in blue) with false discovery ratio (FDR) ≤ 0.05 are differentially increased or reduced by cannabidiol (CBD) relative to control (CON).

Within the amine/phenol analysis, a total of 2,681 unique peak pairs (representing different compounds) were detected. Of those peak pairs, 134 metabolites were positively identified in tier 1 (CIL Library; Supplementary Table 1) and 103 metabolites were putatively identified with high confidence in tier 2 (LI Library; Supplementary Table 2). The PLS-DA scores plot (Figure 1A) shows a clear separation between CON and CBD samples, and the permutation test (*P* < 0.01) confirms the validity of the PLS-DA model (Supplementary Figure 1).

Volcano plot analysis showed that 32 metabolites were differentially altered (FC ≥ 1.2 or ≤ 0.83, FDR ≤ 0.05) by CBD (Figure 1B; Table 1). Eighteen of those metabolites—pyrimidodiazepine, 4-amino-4-deoxychorismate, isoferulic acid, an isomer of D-glucosamine, 7-carboxy-7-carbaguanine, 2,4-dihydroxyhept-2-enedioate, ascorbate, 2′-deamino-2′-hydroxy-6′-dehydroparomamine, trans-2,3-dihydroxycinnamate, gamma-glutamyl-gamma-aminobutyraldehyde, 1,4-diaminobutane, tyramine, an isomer of 2-deoxy-scyllo-inosamine, isoleucyl-alanine, 3-(4-hydroxyphenyl)-pyruvate, aspartyl-threonine, vanillic acid, and D-lysopine—were differentially increased (FC ≥ 1.2, FDR ≤ 0.05) by CBD. The other 14 metabolites—N-acetyl-L-asparagine, alanyl-proline, asparaginyl-aspartic acid, seryl-aspartic acid, phenylalanyl-glycine, prolyl-glutamine, o-tyrosine, N-acetyl-L-adrenaline, L-threo-3-methylaspartate, Z-3-peroxyaminoacrylate, L-glutamate-5-semialdehyde, 2-methyl-3-hydroxy-5-formylpyridine-4-carboxylate, gamma-aminobutyric acid, and aspartyl-glutamine—were differentially reduced (FC ≤ 0.83, FDR ≤ 0.05) by CBD compared to CON.

**Table 1:** Identified amine/phenol-containing metabolites affected by cannabidiol (CBD) compared to control (CON).

**Metabolite**	**Normalized RT^a^**	**FC**	**FDR**	**Identification level^b^**
Pyrimidodiazepine	1,029.2	2.89	0.002	Tier 2
4-Amino-4-deoxychorismate	504.2	2.38	0.004	Tier 2
Isoferulic acid	1,075.1	1.91	0.005	Tier 1
Isomer of D-Glucosamine	152.6	1.85	0.045	Tier 2
7-Carboxy-7-carbaguanine	369.6	1.64	0.019	Tier 2
2,4-Dihydroxyhept-2-enedioate	680.1	1.64	0.004	Tier 2
Ascorbate	530.4	1.62	0.023	Tier 2
2'-Deamino-2'-hydroxy-6'-dehydroparomamine	761.9	1.57	0.026	Tier 2
trans-2,3-Dihydroxycinnamate	856.8	1.53	0.001	Tier 2
gamma-Glutamyl-gamma-aminobutyraldehyde	337.7	1.52	0.010	Tier 2
1,4-Diaminobutane	1,281.7	1.50	0.007	Tier 1
Tyramine	1,538.7	1.50	0.007	Tier 1
Isomer of 2-Deoxy-scyllo-inosamine	208.1	1.47	0.031	Tier 2
Isoleucyl-Alanine	586.8	1.45	0.026	Tier 1
3-(4-Hydroxyphenyl)-pyruvate	1,068.7	1.31	0.019	Tier 2
Aspartyl-Threonine	236.0	1.31	0.019	Tier 1
Vanillic acid	1,026.1	1.26	0.012	Tier 1
D-Lysopine	999.8	1.20	0.019	Tier 2
N-Acetyl-L-Asparagine	492.6	0.83	0.023	Tier 2
Alanyl-Proline	477.2	0.82	0.026	Tier 1
Asparaginyl-Aspartic acid	149.6	0.79	0.026	Tier 1
Seryl-Aspartic acid	162.3	0.78	0.045	Tier 1
Phenylalanyl-Glycine	569.9	0.78	0.026	Tier 1
Prolyl-Glutamine	335.6	0.78	0.012	Tier 1
*o*-Tyrosine	1,313.2	0.77	0.023	Tier 1
N-Acetyl-L-Adrenaline	1,650.1	0.75	0.041	Tier 2
L-threo-3-Methylaspartate	491.9	0.74	0.045	Tier 2
Z-3-Peroxyaminoacrylate	666.3	0.72	0.024	Tier 2
L-Glutamate 5-semialdehyde	367.9	0.72	0.004	Tier 2
2-Methyl-3-hydroxy-5-formylpyridine-4-carboxylate	652.3	0.66	0.032	Tier 2
Aspartyl-Glutamine	194.8	0.65	0.001	Tier 1
Gamma-Aminobutyric acid	466.3	0.55	0.049	Tier 1

*Metabolites with a fold change (FC) ≥ 1.2 relative to CON and a false discovery ratio (FDR) ≤ 0.05 were considered increased by CBD compared to CON. Metabolites with a FC ≤ 0.83 and an FDR ≤ 0.05 were considered reduced in CBD compared to CON*.

a *Normalized RT (retention time) shows the corrected retention time of the peak pair with Universal RT Calibrant data*.

b *Tier 1 indicates positive metabolite identification within the chemical isotope labeling (CIL) metabolite library whereas Tier 2 indicates high confidence putative identification within the linked identity (LI) library*.

**Figure 2:**
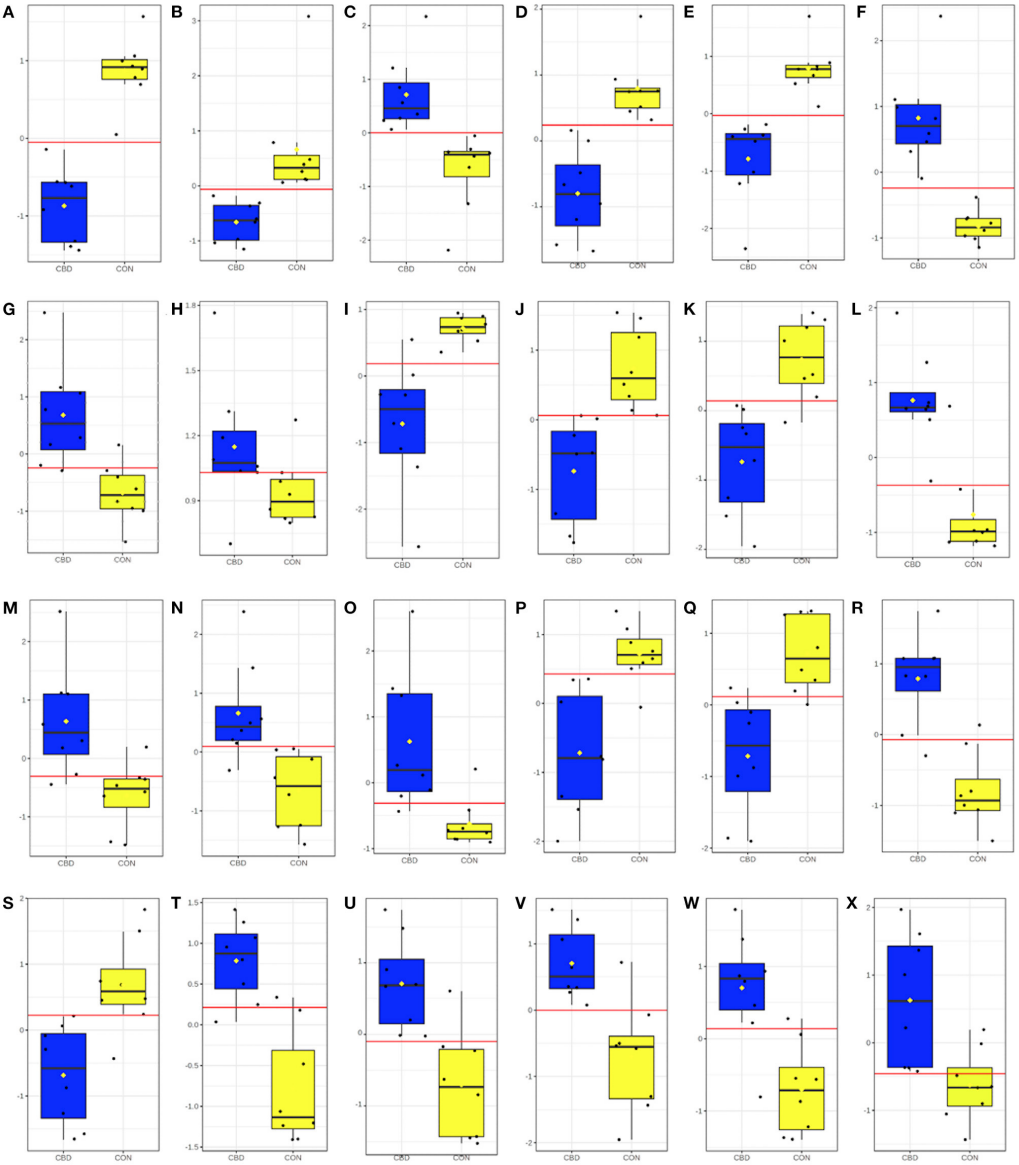
Box and whisker plots for candidate amine/phenol-containing biomarkers altered by cannabidiol (CBD; in blue) compared to control (CON; in yellow). Candidate amine/phenol biomarkers included **(A)** aspartyl-glutamine (AUROC = 1.00; *P* < 0.001); **(B)** gamma-aminobutyric acid (AUROC = 1.00; *P* = 0.005); **(C)** gamma-glutamyl-gamma-aminobutyraldehyde (AUROC = 1.00; *P* < 0.001); **(D)** L-glutamate-5-semialdehyde (AUROC = 1.00; *P* < 0.001); **(E)** prolyl-glutamine (AUROC = 1.00; *P* < 0.001); **(F)** pyrimidodiazepine (AUROC = 1.00; *P* < 0.001); **(G)** 4-amino-4-deoxychorismate (AUROC = 0.98; *P* < 0.001); **(H)** trans-2,3-dihydroxycinnamate (AUROC = 0.98; *P* < 0.001); **(I)** alanyl-proline (AUROC = 0.97; *P* = 0.002); **(J)** N-acetyl-L-asparagine (AUROC = 0.97; *P* < 0.001); **(K)** (Z)-3-peroxyaminoacrylate (AUROC = 0.95; *P* < 0.001); **(L)** 1,4-diaminobutane (AUROC = 0.95; *P* < 0.001); **(M)** 2'-deamino-2'-hydroxy-6'-dehydroparomamine (AUROC = 0.95; *P* = 0.004); **(N)** ascorbate (AUROC = 0.95; *P* = 0.003); **(O)** D-lysopine (AUROC = 0.95; *P* = 0.004); **(P)** *o*-tyrosine (AUROC = 0.95; *P* = 0.001); **(Q)** phenylalanyl-glycine (AUROC = 0.95; *P* = 0.002); **(R)** 2,4-dihydroxyhept-2-enedioate (AUROC = 0.94; *P* < 0.001); **(S)** asparaginyl-aspartic acid (AUROC = 0.94; *P* = 0.003); **(T)** isoferulic acid (AUROC = 0.94; *P* < 0.001); **(U)** 7-carboxy-7-carbaguanine (AUROC = 0.92; *P* = 0.001); **(V)** 3-(4-hydroxyphenyl)pyruvate (AUROC = 0.91; *P* = 0.004); **(W)** aspartyl-threonine (AUROC = 0.91; *P* = 0.005); and **(X)** isoleucyl-alanine (AUROC = 0.91; *P* = 0.007).

Univariate ROC analysis of the 32 identified amine/phenol-containing metabolites that were differentially increased or decreased by CBD revealed 24 metabolites—aspartyl-glutamine, gamma-aminobutyric acid, gamma-glutamyl-gamma-aminobutyraldehyde, L-glutamate-5-semialdehyde, prolyl-glutamine, pyrimidodiazepine, 4-amino-4-deoxychorismate, trans-2,3-dihydroxycinnamate, alanyl-proline, N-acetyl-L-asparagine, (Z)-3-peroxyaminoacrylate, 1,4-diaminobutane, 2′-deamino-2′-hydroxy-6′-dehydroparomamine, ascorbate, D-lysopine, *o*-tyrosine, phenylalanyl-glycine, 2,4-dihydroxyhept-2-enedioate, asparaginyl-aspartic acid, isoferulic acid, 7-carboxy-7-carbaguanine, 3-(4-hydroxyphenyl)-pyruvate, aspartyl-threonine, and isoleucyl-alanine—that appear to be highly predictive of the metabolomic changes between CBD and CON (AUROC ≥ 0.90; *P* < 0.001; Figure 2).

### Carbonyl Metabolites

**Figure 3:**
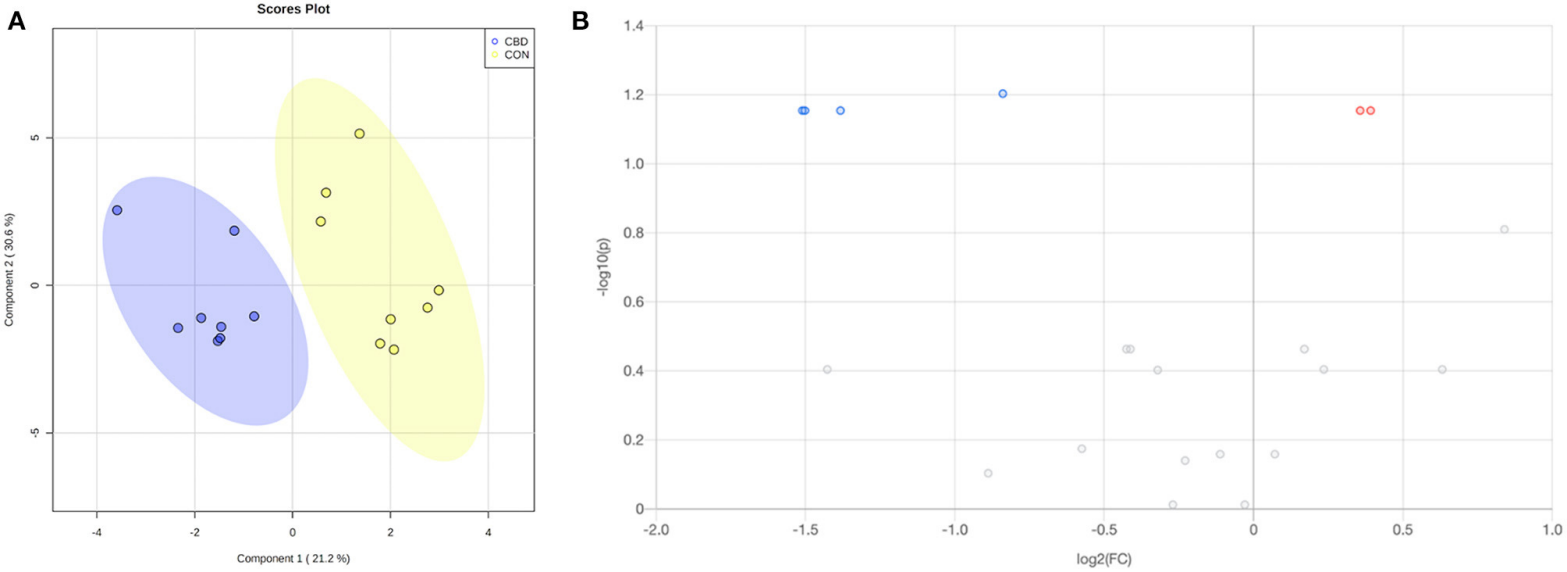
**(A)** Partial least squares discriminant analysis (PLS-DA) scores plot and **(B)** volcano plot showing the differential carbonyl-containing metabolites. Fold change (FC) ≥ 1.2 (in red) or ≤ 0.83 (in blue) with false discovery ratio (FDR) ≤ 0.05 are differentially increased or reduced by cannabidiol (CBD) relative to control (CON).

Within the carbonyl analysis, a total of 612 unique peak pairs were detected. Of those peak pairs, 6 peak pairs were positively identified in tier 1 (CIL Library; Supplementary Table 1) and 15 peak pairs were putatively identified with high confidence in tier 2 (LI Library; Supplementary Table 2). The PLS-DA scores plot (Figure 3A) shows clear separation between CON and CBD samples, and the permutation test (*P* < 0.01) confirms the validity of the PLS-DA model (Supplementary Figure 2).

Volcano plot analysis showed that five metabolites were differentially altered (FC ≥ 1.2 or ≤ 0.83, FDR ≤ 0.05) by CBD (Figure 3B; Table 2). Glucose and 2-formylglutarate were differentially increased (FC ≥ 1.2, FDR ≤ 0.05) by CBD, while glyceraldehyde, isomer of glyceraldehyde, and 4-oxoglutaramate were differentially reduced (FC ≤ 0.83, FDR ≤ 0.05) by CBD compared to control.

**Table 2:** Identified carbonyl-containing metabolites affected by cannabidiol (CBD) compared to control (CON).

**Metabolite**	**Normalized RT^a^**	**FC**	**FDR**	**Identification level^b^**
2-Formylglutarate	569.3	1.99	0.021	Tier 2
Glucose	371.2	1.54	0.018	Tier 1
4-Oxoglutaramate	394.4	0.62	0.050	Tier 2
Isomer of Glyceraldehyde	471.1	0.42	0.035	Tier 1
Glyceraldehyde	453.2	0.38	0.040	Tier 1

*Metabolites with a fold change (FC) ≥ 1.2 relative to CON and a false discovery ratio (FDR) ≤ 0.05 considered increased in the CBD compared to CON. Metabolites with a FC ≤ 0.83 and an FDR ≤ 0.05 considered reduced in CBD compared to CON*.

a *Normalized RT (retention time) shows the corrected retention time of the peak pair with Universal RT Calibrant data*.

b *Tier 1 indicates positive metabolite identification within the chemical isotope labeling (CIL) metabolite library whereas Tier 2 indicates high confidence putative identification within the linked identity (LI) library*.

**Figure 4:**
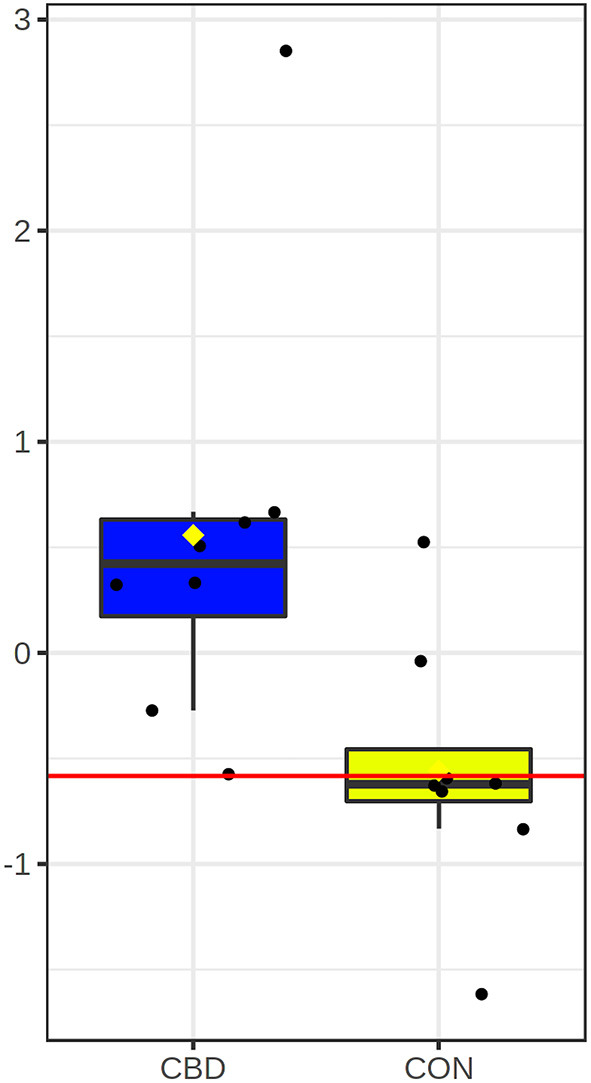
Box and whisker plot for candidate carbonyl-containing biomarker altered by cannabidiol (CBD; in blue) compared to control (CON; in yellow). Glucose (AUROC = 0.91; *P* = 0.020) was the only candidate biomarker for carbonyl-containing metabolites.

Univariate ROC analysis of the five carbonyl metabolites positively and putatively identified that were differentially altered by CBD revealed that plasma glucose appears to be highly predictive of the metabolomic changes between CBD and CON (AUROC = 0.91; *P* = 0.020; Figure 4).

## Discussion

### Amino Acid Metabolism

Increased concentrations of tyramine, 3-(4-hydroxyphenyl)-pyruvate, 2,4-dihydroxyhept-2-enedioate, gamma-glutamyl-gamma-aminobutyraldehyde, 1,4-diaminobutane, and D-lysopine may indicate that CBD altered amino acid metabolism. Tyramine, 3-(4-hydroxyphenyl)-pyruvate, and 2,4-dihydroxyhept-2-enedioate are intermediates in tyrosine and phenylalanine metabolism (32–34). Tyramine, in particular, is also involved in the biosynthesis of many secondary metabolites in plants, such as isoquinoline alkaloids, flavonoids, and hydroxycinnamic acid amines (35, 36). Gamma-glutamyl-gamma-aminobutyraldehyde and 1,4-diaminobutane (i.e., putrescene) are intermediates in arginine, proline, and ornithine degradation pathways (37, 38). Putrescene is also known to play a role in the regulation of cell growth, protein synthesis, apoptosis, and other cellular processes (39, 40). D-lysopine is an amino opine derivative of L-lysine found in crown gall tumors produced by pathogenic bacteria that infect plants, including *C. sativa*. While not known to be produced in mammalian systems, other opines like saccharopine are known intermediates in the metabolism of lysine in mammals (41–43). The increase in these metabolites may suggest that CBD enhanced degradation of these amino acids.

Decreased concentrations of N-acetyl-L-asparagine, *o*-tyrosine, N-acetyl-L-adrenaline, L-threo-3-methylaspartate, L-glutamate-5-semialdehyde, and 4-oxoglutaramate may also suggest that CBD altered metabolism of other amino acids. N-acetyl-L-asparagine is a derivative of asparagine that is N-acetylated by N-acetyltransferase 1 (NAT1), one of several acetyltransferases known to play a role in drug metabolism (44). This enzyme has been suggested to play a role in the regulation of mTOR complex I activation, cancer cell proliferation, and mitochondrial function (44–46). As CBD is suspected to exert anti-cancer effects, it may be prudent in future work to investigate if CBD supplementation alters NAT1 activity. *o*-Tyrosine is a structural isomer of tyrosine and a phenylalanine derivative. It is considered a marker for oxidative stress as it is produced through free-radical hydroxylation of phenylalanine (47, 48). If the decrease in *o*-tyrosine was due to CBD supplementation, this may contribute to the suspected antioxidative effects of CBD. N-acetyl-L-adrenaline is a methylated form of epinephrine, an adrenal hormone involved in the regulation of visceral functions (49, 50).

L-threo-3-methylaspartate is an amino acid formed by glutamate mutase and can be metabolized by methylaspartate ammonia-lyase. It is found in the structures of the antibiotics friulimicin and vicenistatin and in carbon metabolism of haloarchaea (51, 52). L-glutamate-5-semialdehyde is a non-proteinogenic amino acid that is an intermediate in both proline and arginine biosynthesis from glutamate (53, 54). 4-oxoglutaramate is an intermediate in one of the histidine catabolism pathways that leads to the production of 2-oxoglutarate (i.e., α-ketoglutarate), which then feeds into the citric acid cycle (55, 56). Aspartyl-glutamine, aspartyl-threonine, alanyl-proline, asparaginyl-aspartic acid, isoleucyl-alanine, phenylalanyl-glycine, prolyl-glutamine, and seryl-glycine are products of the incomplete breakdown of protein digestion or catabolism. While some dipeptides are known to have physiological or cell-signaling effects, none of the affected dipeptides have been identified as one of these bioactive molecules (57, 58).

The altered concentrations of these metabolites suggest an effect of CBD on amino acid metabolism. However, since the relative concentrations of the individual amino acids (arginine, aspartate, glutamate, and proline) were unaffected by treatment, the biological significance of the changes in these metabolites is unclear. Additional research is needed to assess the potential for CBD to alter amino acid metabolism.

### Glucose Metabolism

The increase in glucose, an isomer of glucosamine, 2′-deamino-2′-hydroxy-6′-dehydroparomamine, and an isomer of 2-deoxy-schyllo-inosamine may suggest that glucose metabolism was altered by CBD. The endocannabinoid system—by which CBD and other cannabinoids exert physiological effects—plays a well-established role in glucose and energy metabolism, marking it as a target for the treatment of metabolic diseases like type 2 diabetes (59). Cannabinoids like CBD and THC have been suggested to reduce hyperglycemia and increase insulin production in rodents (60, 61), but this has yet to be investigated in a canine model.

Glucosamine is an amino sugar that is readily synthesized in the body from glucose and glutamine. It is an essential component of mucopolysaccharides that are incorporated into connective tissue, mucous secretions, skin, tendons, ligaments, and cartilage. Additionally, it helps regulate the synthesis of collagen in cartilage (62–64). Because of its high concentration in joint tissues, glucosamine is commonly used as a dietary supplement in humans, horses, and dogs as a support for joint health and function and to relieve symptoms of osteoarthritis, though there is little scientific evidence supporting these effects (65). Both 2-deoxy-scyllo-inosamine and 2'-deamino-2'-hydroxy-6'-dehydroparomamine are intermediates in the biosynthesis of aminoglycoside antibiotics, like kanamycin, from glucose in Streptomyces bacterial species (66, 67). However, since these metabolites are not known to be generated in mammalian systems, the biological significance of this is unclear.

The decrease in gamma-aminobutyric acid (GABA), glyceraldehyde, and an isomer of glyceraldehyde may also indicate that CBD altered glucose metabolism. Best known as the primary inhibitory neurotransmitter in the central nervous system, GABA is also produced by insulin-producing β cells of the pancreas and immune cells (68, 69). In the pancreas, GABA inhibits glucagon secretion from neighboring α cells and modulates glucose homeostasis (68, 70, 71). This action of GABA in the pancreas has highlighted its potential as a target for diabetes treatment (72). It has also been shown to regulate cytokine secretion from human peripheral blood mononuclear cells (PBMCs) and CD4+ T cells and is thought to exert anti-inflammatory effects (73–75).

Glyceraldehyde, a triose monosaccharide, is an intermediate in glycolysis in its phosphorylated form (GAP). Glyceraldehyde-3-phosphate dehydrogenase (GAPDH), the enzyme that catalyzes the conversion of GAP into 1,3-bisphosphoglycerate, is a major regulator of carbon flux in the body (76). It is also known to play a role in other cellular functions such as redox sensing, membrane fusion, iron homeostasis, and cell death (77). The decrease in glyceraldehyde may lend support to the suspected anti-obesity and anti-diabetic effects of CBD; however, the increase in glucose and decrease in GABA would appear to be incongruous with this potential effect. These results highlight a relatively unexplored avenue of CBD research that warrants further investigation.

### Hydroxycinnamic Acid Derivatives

The increase in plasma isoferulic acid (IFA), trans-2,3-dihydroxycinnamate, and vanillic acid may suggest that CBD altered the metabolism of hydroxycinnamic acid derivatives. Isoferulic acid is a naturally occurring hydroxycinnamic acid derivative commonly found in Lobelia and Cimicifuga species. It is an isomer of ferulic acid, a phenolic compound that is a component of lignin, which is commonly found in cell walls of plants, including C. sativa (78). Ferulic acid has also been isolated from hemp seed meal, a byproduct of hemp oil processing (79). Both ferulic acid and IFA have been reported to have anti-inflammatory, anti-viral, anti-oxidative, and anti-diabetic properties and are commonly used as ingredients in herbal medicines in Japan and China (80, 81). Isoferulic acid has been shown to reduce plasma glucose in diabetic rats and to inhibit IL-8 production in mice (82, 83). Additionally, IFA has been suggested to act as an anti-glycation compound. Protein glycation is a non-enzymatic reaction associated with oxidative stress and reactive oxygen species (ROS) production; it is thought to be a contributor to age-related diseases (84). In several studies, IFA protected against fructose- and glucose-mediated glycation and inhibited ROS production *in vitro* (80, 85). In humans, IFA has been shown to be an intermediate in the metabolism of plant-derived phenolic compounds like caffeic acid (86), with humans obtaining the majority from dietary consumption.

Trans-2,3-dihydroxycinnamate is a derivative of cinnamic acid, which is an intermediate in the biosynthesis of lignin, flavonoids, and other secondary metabolites produced by plants like *C. sativa* (87). Cinnamic acid and its derivatives, like trans-2,3-dihydroxycinnamate, possess antioxidizing activity (88). Vanillic acid is a dihydroxybenzoic acid derivative commonly used as a flavoring agent. It is also an intermediate in the synthesis of vanillin from ferulic acid and a phenolic compound that, like IFA, is a component of lignin present in the secondary cell wall of plants, including *C. sativa* (89, 90). Like other lignin-associated aromatic acids, vanillic acid has been reported to exert antimicrobial properties (91). If the increase in these metabolites is a result of CBD supplementation, it is possible that these compounds may contribute to the suspected anti-microbial, anti-inflammatory, and antioxidative effects of hemp. However, it is unlikely that the changes in these compounds are solely due to increased dietary consumption as the treats used in this study provided a small quantity of industrial hemp extract. Conversely, it may be possible that changes in the metabolism of these hydroxycinnamic acid derivatives occurred as a result of CBD; additional work is warranted to further investigate these potential effects.

### Vitamin and Nucleotide Metabolism

Increased concentrations of pyrimidodiazepine, 4-amino-4-deoxychorismate, 7-carboxy-7-carbaguanine, 2-formylglutarate, and ascorbate may indicate an alteration of vitamin and nucleotide metabolism. Pyrimidodiazepine is a derivative of uracil and a substrate for pyrimidodiazepine synthase, an enzyme that can contribute to glutathione synthesis. Uracil derivatives like pyrimidodiazepine are also thought to possess antimicrobial and antioxidant properties (92). 4-amino-4-deoxychorismate is a precursor for para-aminobenzoic acid (pABA) biosynthesis, which is a precursor for folic acid (vitamin B9) synthesis in plants and microorganisms (93, 94). While vitamin B9 is an essential cofactor that facilitates methyl transfers, mammals do not possess the enzymes to produce folic acid and instead rely on dietary consumption of the vitamin (93). 7-carboxy-7-carbaguanine is pyrrolopyrimidine that, like 4-amino-4-deoxychorismate, is involved in the biosynthesis of vitamin B9 (95). 2-formylglutarate is an intermediate in nicotinamide metabolism in several bacterial species [(96, 97)]. Ascorbate, or vitamin C, can be synthesized in dogs from glucose or ingested in the diet (98, 99). Vitamin C serves as a cofactor in several essential reactions, including collagen synthesis and wound healing, as well as an antioxidant. If the increase in ascorbate was due to CBD supplementation, this may contribute to the suspected antioxidative effects of CBD.

Decreased concentrations of 2-methyl-3-hydroxy-5-formylpyridine-4-carboxylate and (Z)-3-peroxyaminoacrylate may also support an effect of CBD on vitamin and nucleotide metabolism. 2-methyl-3-hydroxy-5-formylpyridine-4-carboxylate is an intermediate in the pyridoxine (vitamin B6) degradation pathway (100). Vitamin B6 is an essential cofactor in several enzymatic reactions, including the synthesis of glutathione, an important antioxidant (101). (Z)-3-peroxyaminoacrylate is an intermediate in bacterial pyrimidine degradation pathway known as the Rut pathway. However, this pathway and intermediate are not known to play a role in mammalian pyrimidine metabolism (102).

Since the relative concentrations of pyridoxine, uracil, and folate were not affected by treatment, and since several of these metabolites are not known to be generated in mammalian systems, the biological significance of these changes is unclear. However, increased pyrimidodiazepine and ascorbate, along with decreased 2-methyl-3-hydroxy-5-formylpyridine-4-carboxylate, may indicate an influence of CBD on antioxidant status. Further studies are needed to determine the roles of these metabolites and the potential effects of CBD on these pathways.

### Strengths and Limitations

Cannabidiol is already being supplemented to dogs for its potential therapeutic applications including osteoarthritis, separation anxiety, noise phobias, and epilepsy (4, 11). Several studies have evaluated its effectiveness in dogs with osteoarthritis (23, 103, 104) noise phobias (105), and epilepsy (106) with mixed results. Considerable work has also been done investigating pharmacokinetics (21–23, 107) and safety (108, 109) following oral CBD administration; however, this study is the first to evaluate the impact of CBD supplementation on the canine metabolome. This analysis provides a comprehensive scan of potential metabolic targets in dogs receiving CBD; however, because of the lack of metabolic profile with CBD it was not intended to be all-encompassing but rather a first look into the potential for CBD supplementation to alter the canine metabolome. Thus, this study may be limited by the relatively short duration of CBD supplementation, small sample size, lack of baseline measurement, and the use of only a single CBD dosage. Even so, identifying changes in the metabolome is essential for directing future targeted investigations into both the physiological relevance of these changes as well as elucidating potential mechanisms leading to these observed effects. It would be beneficial for future work to evaluate metabolomic changes in an unhealthy or diseased population of dogs supplemented with CBD and potential differences between acute and long-term CBD administration.

## Conclusions

This study demonstrated the canine metabolome was altered with 4.5 mg CBD/kg BW/d supplementation for 3 weeks. Altered metabolites may suggest a potential for CBD to influence glucose, amino acid, vitamin, and nucleotide metabolism. Additionally, the increase in relative concentrations of metabolites like *o*-tyrosine, IFA, glucosamine, and pyrimidodiazepine may indicate potential pathways by which CBD may exert suspected anti-inflammatory, antioxidant, and antimicrobial effects. Several metabolites were identified as potential biomarkers for changes in the canine metabolome by CBD. Further studies with larger sample sizes, longer supplementation periods and baseline comparisons to refine metabolites are necessary to elucidate the physiological relevance of these changes.

## Data Availability Statement

The original contributions presented in the study are included in the article/Supplementary Material, further inquiries can be directed to the corresponding author/s.

## Ethics Statement

The animal study was reviewed and approved by LMU IACUC. Written informed consent was obtained from the owners for the participation of their animals in this study.

## Author Contributions

DH, EM, KM, and SK-M contributed to the conception and design of the study. EM, SK-M, and DS facilitated data collection. EM and IO performed statistical analysis. EM wrote the first draft of the manuscript. All authors contributed to manuscript revision, read, and approved the submitted version.

## Conflict of Interest

The authors declare that the research was conducted in the absence of any commercial or financial relationships that could be construed as a potential conflict of interest.

## Supplementary Material

Supplementary Table 1 Amine/phenol- and carbonyl-containing metabolites identified in the tier 1 chemical isotope labeling (CIL) library.

Supplementary Table 2 Amine/phenol- and carbonyl-containing metabolites identified in the tier 2 linked identity (LI) library.

Supplementary Figure 1 Partial least squares discriminant analysis (PLS-DA) model permutation for amine/phenol-containing metabolites.

Supplementary Figure 2 Partial least squares discriminant analysis (PLS-DA) model permutation for carbonyl-containing metabolites.
