# Supplementary figures and images for: Alteration of the Canine Metabolome After a 3-Week Supplementation of Cannabidiol (CBD) Containing Treats: An Exploratory Study of Healthy Animals
Source: Front Vet Sci. 2021 Jul 16;8:685606. doi: 10.3389/fvets.2021.685606 (PMC8322615; doi:10.3389/fvets.2021.685606)

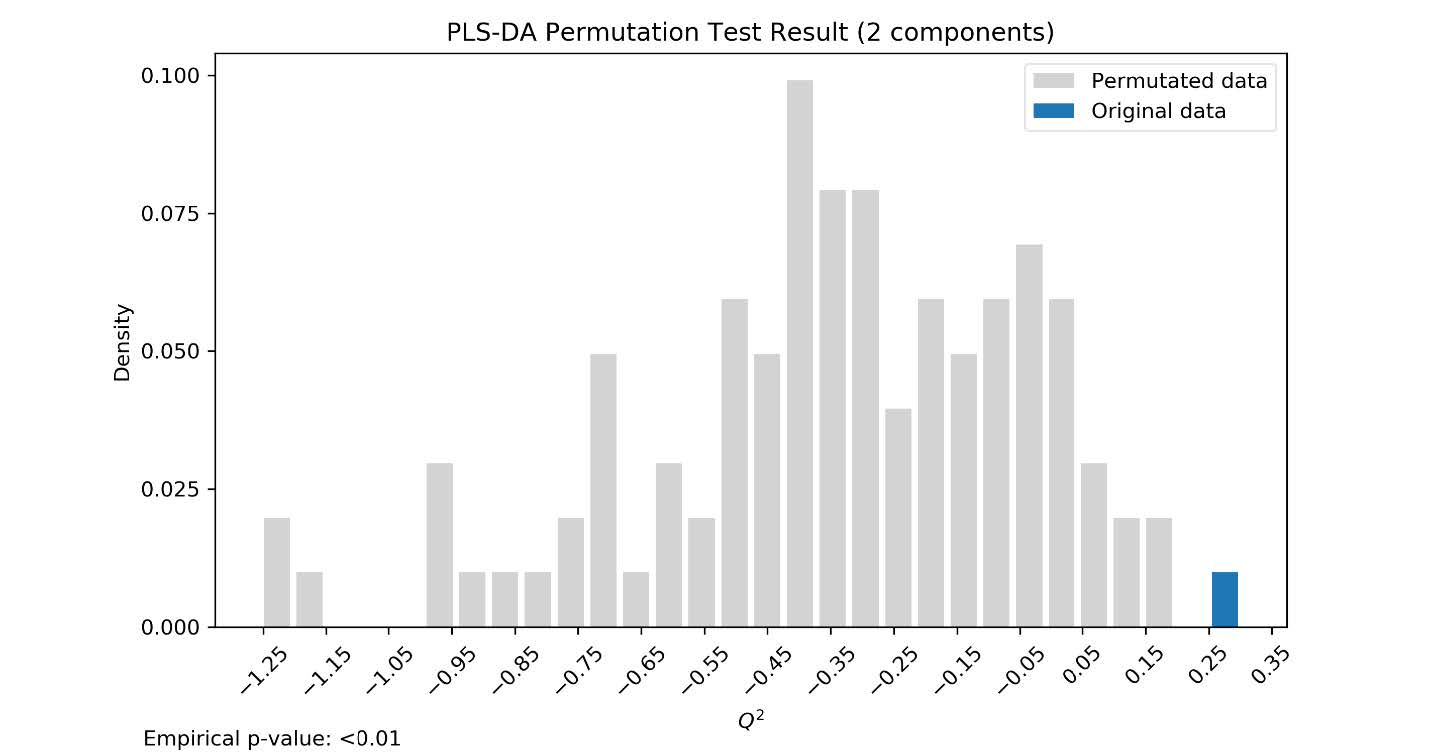

Supplement: Supplementary Figure 1 — Partial least squares discriminant analysis (PLS-DA) model permutation for amine/phenol-containing metabolites. [file Image_1.TIFF]

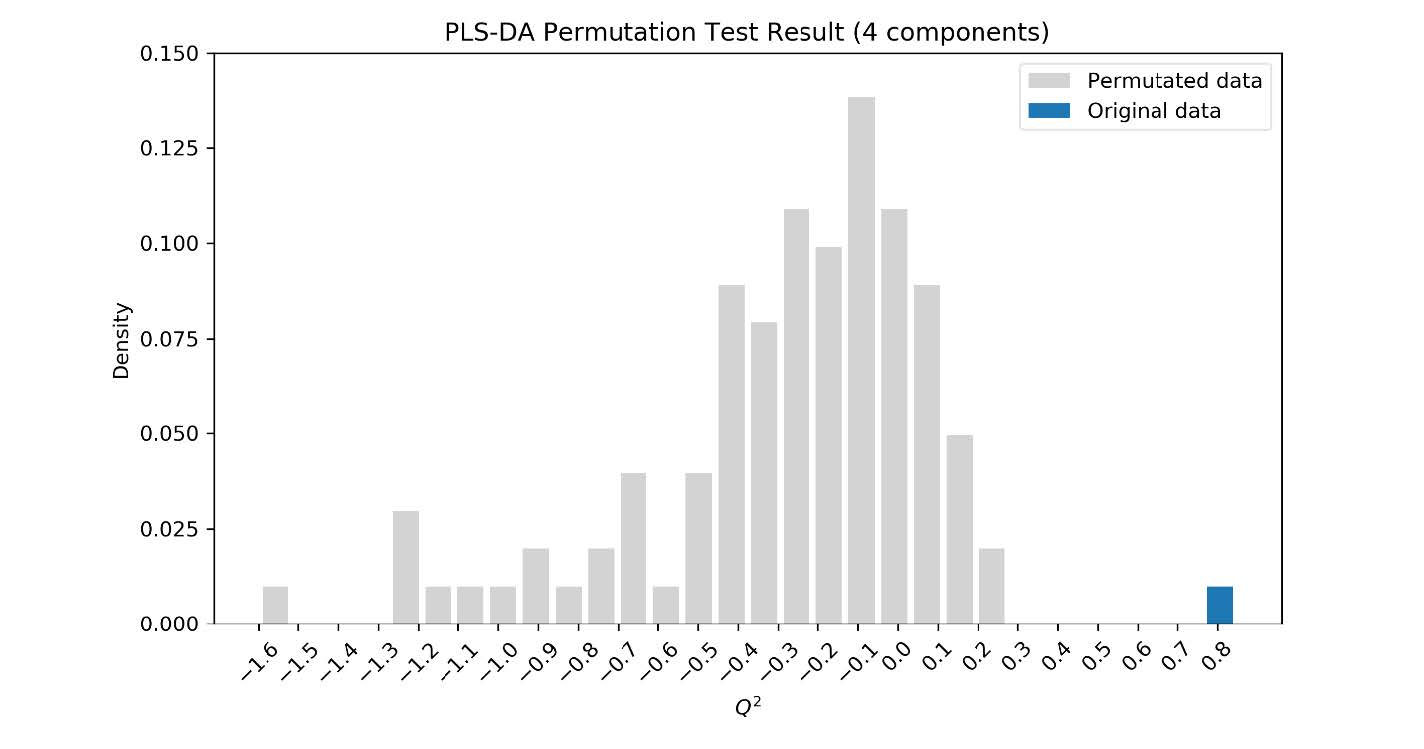

Supplement: Supplementary Figure 2 — Partial least squares discriminant analysis (PLS-DA) model permutation for carbonyl-containing metabolites. [file Image_2.TIFF]
